# Commentary: A comparative study on closed reduction vs. open reduction: Techniques in the surgical treatment of rotated lateral condyle fractures of the distal humerus in children

**DOI:** 10.3389/fped.2022.1056128

**Published:** 2022-11-23

**Authors:** Andreas Rehm, Elizabeth Ashby, Pinelopi Linardatou Novak

**Affiliations:** Department of Paediatric Orthopaedics, Cambridge University Hospitals NHS Trust, Cambridge, United Kingdom

**Keywords:** lateral condyle fracture, milch classification, jakob classification, CRPP, closed reduction and percutaneous pinning, humerus fracture, pediatric elbow trauma, song classification

A Commentary on A comparative study on closed reduction vs. open reduction: Techniques in the Surgical treatment of rotated lateral condyle fractures of the distal humerus in children By Weng L, Cao Y, Zhang G, Zhou H, Liu X, Zhang Y. (2022). Front. Pediatr. 10:891840. doi: 10.3389/fped.2022.891840

We read with interest the article by Weng et al. Neither the Jakob (1) nor the Song (2) classification considers the anatomic variations of lateral humeral condyle fractures (LHCF). Both (1, 2) do not differentiate between Milch (3) type I fractures (fracture line runs through the capitello-trochlear sulcus or lateral to it) and type II fractures (fracture line runs through the trochlea). Song et al.'s (2) illustration of stage 1 to 5 fractures depicts only Milch type II fractures of increasing severity, which only applies to avulsion fractures caused by forearm adduction injuries. These limitations have possibly resulted in the classification having been abandoned by Song et al. (4) two years after its publication.

Weng et al. included only Song stage 5 fractures which are the same as Jakob type III (displaced and rotated fragment) and did not differentiate between Milch type I and II.

Xie et al. (5) reported an overall closed reduction and percutaneous pinning (CRPP) rate of 74% for LHCFs with >4 mm displacement. There was no difference in the CRPP rate between Song stage 4 (75%; 15 of 20 cases) and stage 5 cases (73%; 22 of 30 cases) but there was a significant difference between Milch type I (50%; 6 of 12 cases) and II (82%; 31 of 38 cases) fractures. All 30 Song stage 5 patients had an initial attempt of closed reduction. In 11 of the latter patients a 2 mm K-wire was used as a joystick which resulted in a closed reduction in 6 patients but in 5 it had to be proceeded to an open reduction. Information on the length of the individual procedures was not provided. Xie et al. (5) concluded that a closed reduction should always be attempted and that the fracture anatomy, as identified by the Milch classification, is more important for the success rate than the Song classification. We would like to ask Weng et al. if they could identify the Milch types for their fractures and if there was an association between Milch type I and increased ORPP rate?

Most of the fracture healing happens within the bone and is in our opinion impossible to measure or judge accurately, so that we generally leave children in a cast for about 5 weeks and then take radiographs after cast removal. It would have been necessary for Weng et al. to have had a fixed follow-up and clearly defined bone healing assessment protocol (which does not exist for these fractures) to identify a difference in the fracture healing time between the groups, with cast removal, taking of radiographs and cast re-application (where necessary) on a weekly basis from 4 weeks until it was judged for the fractures to have healed. Since the authors did not describe such protocol, we assume that the casting times and reported bone healing times were purely dependent on the surgeon's preference, with the different bone healing times only reflecting the arbitrary choice of casting times. Do Weng et al. agree that their study design and provided evidence does not support their statement that CRPP is associated with a reduced bone healing time compared to open reduction?

We would also like to ask Weng et al. how they explain their high superficial (x5) and deep (x2) infection rate in their open reduction and percutaneous pinning group (ORPP) in comparison to Nazareth et al. (6) who reported 1 superficial and no deep infection in 30 patients with >4 mm displacement who had ORPP. Deep infections create a lot of hardship for the children and their parents, requiring intravenous antibiotics *via* a PICC-/long line and sometimes wound and/or joint washout. This creates a lot of costs for the health provider which might outweigh the costs for the extended operating time needed for CRPP reported by Weng et al.

Weng et al. reported that CRPP of Song stage 5 LHCFs was generally difficult, not possible in 33% of cases and was associated with a time-consuming learning process and therefore identified open reduction and fixation as their “gold standard”, indicating that CRPP is a technique which requires acquired experience and skills and should probably be left to those who perform such procedures regularly.

In conclusion, Weng et al. identified that CRPP of LHCF is technically difficult but the data provided by the latter and other authors (1, 2) support that closed CRPP of LHCF displaced >4 mm is possible in a high proportion of fractures with good outcomes. Therefore, the way forward might be to attempt CRPP of all fractures, considering the high infection rate reported by Weng et al. for ORPP (19.4%), the larger scars from ORPP and the very low infection rate reported by Bloomer et al. (7) for CRPP of supracondylar humerus fractures with (0.6%) and without antibiotic (0.4%).
